# Strategies to overcome HIV drug resistance-current and future perspectives

**DOI:** 10.3389/fmicb.2023.1133407

**Published:** 2023-02-16

**Authors:** Aura Temereanca, Simona Ruta

**Affiliations:** ^1^Virology Department, Carol Davila University of Medicine and Pharmacy, Bucharest, Romania; ^2^Viral Emerging Diseases Department, Stefan S. Nicolau Institute of Virology, Bucharest, Romania

**Keywords:** HIV infection, drug resistance, newly approved antiretroviral agents, salvage therapy, new drug targets, patients with multidrug-resistant HIV-1 infection

## Abstract

The availability of combined antiretroviral therapy (cART) has revolutionized the course of HIV infection, suppressing HIV viremia, restoring the immune system, and improving the quality of life of HIV infected patients. However, the emergence of drug resistant and multidrug resistant strains remains an important contributor to cART failure, associated with a higher risk of HIV-disease progression and mortality. According to the latest WHO HIV Drug Resistance Report, the prevalence of acquired and transmitted HIV drug resistance in ART naive individuals has exponentially increased in the recent years, being an important obstacle in ending HIV-1 epidemic as a public health threat by 2030. The prevalence of three and four-class resistance is estimated to range from 5 to 10% in Europe and less than 3% in North America. The new drug development strategies are focused on improved safety and resistance profile within the existing antiretroviral classes, discovery of drugs with novel mechanisms of action (e.g., attachment/post-attachment inhibitors, capsid inhibitors, maturation inhibitors, nucleoside reverse transcriptase translocation inhibitors), combination therapies with improved adherence, and treatment simplification with infrequent dosing. This review highlight the current progress in the management of salvage therapy for patients with multidrug-resistant HIV-1 infection, discussing the recently approved and under development antiretroviral agents, as well as the new drug targets that are providing a new avenue for the development of therapeutic interventions in HIV infection.

## Introduction

**Figure 1:**
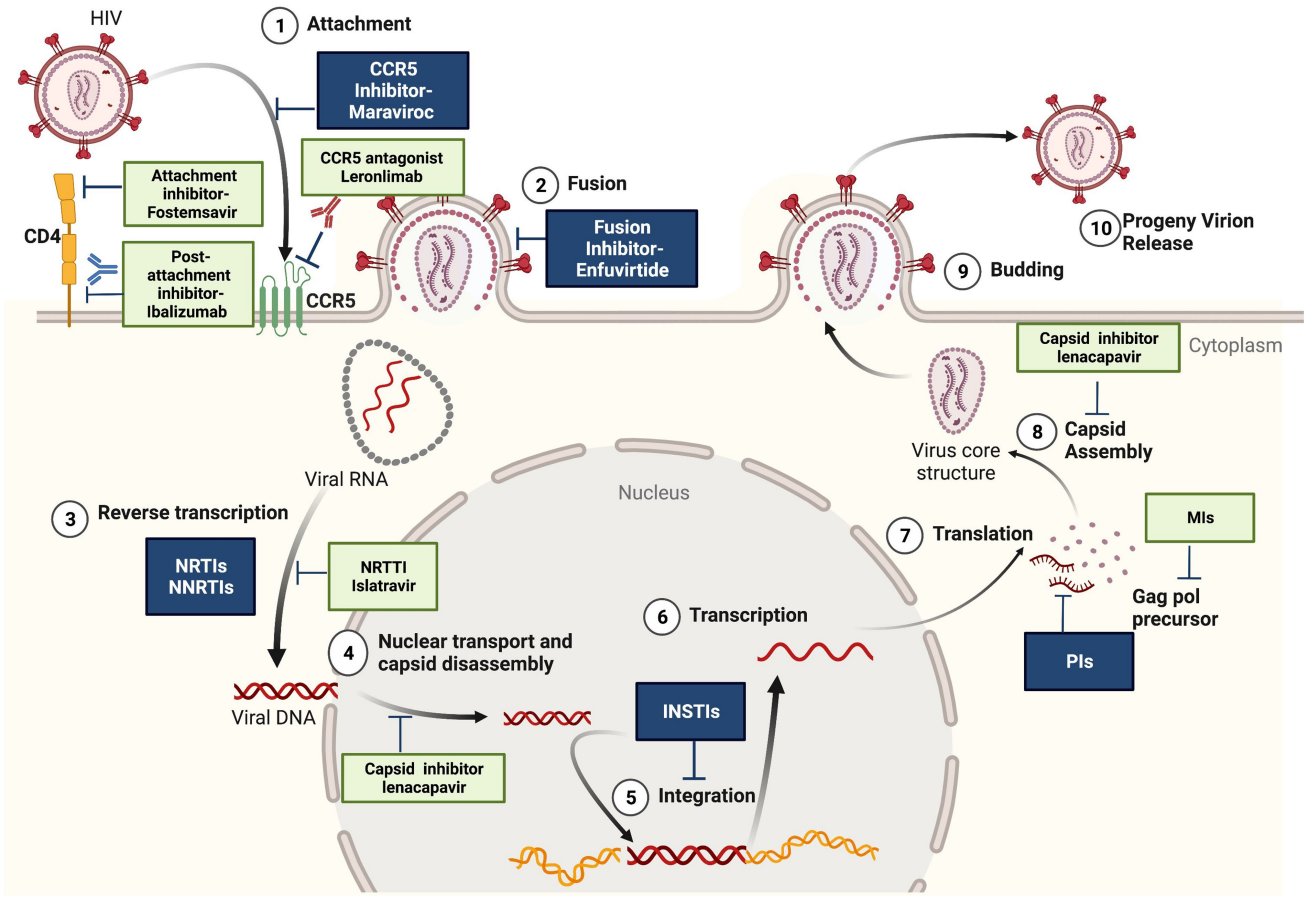
Antiretroviral agents. In dark-the conventional ART used in combinations: entry inhibitors, reverstranscription inhibitors (NRTI-nucleoside analogs reverstranscriptase inhibitors and NNRTI-non-nucleoside reverstranscriptase inhibitors), INSTI-integrase inhibitors, PI-protease inhibitors. In light color-newly approved antiretrovirals-fostemsavir (CD4 attachement inhibitor), ibalizumab (mAb gp120 post attachment inhibitor), lenacapavir (capsid inhibitor) and antiretrovirals in advanced clinical trials: leronlimab (mAb CCR5 antagonist), islatravir (NNRTTI-nucleoside reverstranscriptase translocation inhibitor) and maturation inhibitors. Created with BioRender.com, with a license to publish.

In 2021, 38.4 million people were living with HIV infections and 1.5 million people became newly infected with HIV according to the recent update from The Joint United Nations Programme on HIV/AIDS.^1^ The availability of combined antiretroviral therapy (cART) has revolutionized the course of HIV infection, suppressing HIV viremia, restoring the immune system, and improving the quality of life of HIV infected patients (Samji et al., 2013). Current HIV treatment includes five different classes of antiretrovirals targeting multiple steps of the HIV life cycle (Figure 1): entry inhibitors that block (1) the attachment of HIV envelope glycoprotein gp120 to CCR5 co-receptors (maraviroc) or (2) the cell fusion mediated by HIV gp41 (enfuvirtide), (3) nucleoside reverse transcriptase inhibitors (NRTIs) and non-nucleoside reverse transcriptase inhibitors (NNRTIs) that block the reverse transcription of viral RNA to cDNA, (4) integrase strand transfer inhibitors (INSTIs) that inhibit the integration of proviral DNA into host genome, and (5) protease inhibitors (PIs) that inhibit the protease-mediated cleavage of gag and gag-pol precursors, resulting in the production of non-infectious virus particles (De Clercq and Li, 2016). However, the emergence of drug resistant and multidrug resistant strains remains an important contributor to cART failure, associated with a higher risk of HIV-disease progression and mortality (Galli et al., 2020). Cross-resistance between drugs within the same class can occur and affects all major classes of antiretroviral drugs (Puertas et al., 2020). According to the latest WHO HIV Drug Resistance Report, the prevalence of acquired and transmitted HIV drug resistance in ART naive individuals has exponentially increased in the recent years, being an important obstacle in ending HIV-1 epidemic as a public health threat by 2030.^2^ The report indicates that 10% of adults starting HIV treatment have resistance to non-nucleoside reverse transcriptase inhibitors (NNRTIs) and people with previous exposure to antiretroviral drugs are three times more likely to present resistance to the NNRTI drug class. The prevalence of three and four-class drug-resistant viruses is estimated to be 5 to 10% in different cohorts of antiretroviral-experienced patients from Europe and less than 3% in North America (Judd et al., 2017; Galli et al., 2020; Puertas et al., 2020; Lombardi et al., 2021). Global efforts are required to design effective strategies to address HIV drug resistance: routine viral load monitoring, improving adherence to treatment, conducting drug resistance genotyping, regimen switch if indicated and selection of the most effective antiretroviral drugs combinations to achieve l*ong*-*term* and successful *treatment outcomes*. Two or three fully active agents are recommended to construct a new regimen for highly treatment-experienced patients with multidrug-resistant virus.^3^ Therefore, the development of new drugs is focused on (a) improved safety and resistance profile within the existing antiretroviral classes, (b) combination therapies with improved adherence, (c) treatment simplification with infrequent dosing, using long-acting agents to overcome the adherence issues and prevent the development of drug resistance (Overton et al., 2020; Flexner et al., 2021) and (d) discovery of drugs with novel mechanisms of action (e.g., attachment/post-attachment inhibitors, capsid inhibitors, maturation inhibitors, nucleoside reverse transcriptase translocation inhibitors).

This review highlight the current progress in the management of salvage therapy for patients with multidrug-resistant HIV-1 infection, discussing antiretroviral agents that are recently approved and those under development, as well as the new drug targets providing a different avenue for the development of therapeutic interventions in HIV infection.

## Recently approved and in clinical development antiretroviral agents for the treatment of drug resistant HIV-1

Novel types of antiretrovirals with new mechanisms of action have been developed in the recent years, offering additional therapeutic options for patients with multiclass drug-resistant HIV infection (Figure 1).

### Fostemsavir-CD4 attachment inhibitor

Fostemsavir (FTR) is the first FDA-approved attachment inhibitor indicated for heavily treated adults with multidrug-resistant HIV-1 (Gulick, 2018). FTR is a prodrug of the active molecule temsavir, which inhibits the HIV-1 gp 120 conformational changes needed for CD4 attachment, preventing viral entry into susceptible cells (Cahn et al., 2018). The efficacy and safety of fostemsavir has been evaluated in a partially randomized, double-blind, placebo-controlled clinical trial (BRIGHTE), which enrolled 371 highly treatment-experienced patients who have failed their current regimen (Kozal et al., 2020; Lataillade et al., 2020). FTR showed sustained rates of virologic suppression over the 96 weeks of clinical trial: the proportion of participants who achieved undetectable HIV viral load was 53% at week 24, 54% at week 48, and 60% at week 96 and CD4^+^ T-cell recovery was recorded even in the most immunocompromised patients (participants with less than 20 cells/mm^3^ at baseline had a mean CD4^+^ T-cell increase of 240 cells/mm^3^ at week 96) (Ackerman et al., 2021).

The most commonly reported adverse effects for FTR included nausea, vomiting, diarrhea, and headache. *Temsavir* is partly *metabolized by CYP3A4* enzyme, and it is contraindicated with any strong CYP3A4 inducers (carbamazepine, rifampin) (Hiryak and Koren, 2021).

#### Resistance

A study published in 2020 analyzed HIV-1 *env* gp120 sequences from both ART-naïve and ART-treated patients and identified several genomic positions with mutations associated with decreased susceptibility to fostemsavir (Bouba et al., 2020), however, the BRIGHTE trial did not find consistent associations between virologic failure and gp120 substitutions (Kozal et al., 2020; Lataillade et al., 2020).

No cross-resistance with other antiretroviral agents have been reported (Rose et al., 2022), fostemsavir being considered a life-saving option for patients with drug-resistant HIV.

### Antibody-based strategies

Monoclonal antibodies are becoming attractive strategies for HIV treatment, with good resistance profile, ability to restore CD4 T-cell count and lack of toxicity.

### Ibalizumab- post-attachment inhibitor

Ibalizumab (IBA)- the first monoclonal antibody approved for the treatment of HIV-1 infection (Markham, 2018)- is a long-acting post-attachment inhibitor, which binds to the extracellular CD4 domain, leading to conformational changes of the CD4 T cell receptor–gp120 complex and blocking HIV entry (Chahine and Durham, 2021). Importantly, the binding of IBA to CD4 cell receptors does not induce an immunosuppressive response. Ibalizumab, in combination with other antiretrovirals, is indicated for the treatment of heavily treatment-experienced adults with multidrug-resistant HIV-1 infection with limited or no other treatment options (Markham, 2018) and it is designed for *twice*-*monthly intravenous administration* (Chahine and Durham, 2021).

The approval of ibalizumab was based on a phase III, single-group, open-label study (TMB-301), that enrolled 40 heavily treatment-experienced HIV patients who had failed several antiretroviral regimens, had >1,000 copies/mL HIV viral load and resistance to at least one NRTI, NNRTI, and a PI (Emu et al., 2018). The results showed that ibalizumab significantly decreased the viral load 7 days after being added to the failing antiretroviral regimen. Furthermore, ibalizumab plus an individually optimized background regimen significantly reduced the HIV-1 viral load at week 25, viral suppression being reported in 43% of the study participants and increased the CD4 T cell count with a mean of 62 cells/mm^3^.

Ibalizumab was well tolerated in the TMB-301 clinical trial, the most common adverse reactions included diarrhea, dizziness, nausea, and rash. There were no significant drug–drug interactions or adverse interactions between ibalizumab and other antiretroviral agents.

#### Resistance

Resistance to ibalizumab is conferred by decreased viral expression of specific binding sites in the HIV gp120 envelope protein (Pace et al., 2013). This mechanism of resistance was observed in the TMB-301 study for 8 out of 10 patients who had virologic failure or rebound at week 25 and showed a lower degree of susceptibility to ibalizumab than at baseline (Emu et al., 2018). Currently, there are no commercially available resistance testing methods for patients with suspected resistance to IBA (Blair, 2020).

No cross resistance was reported between ibalizumab and all the other approved antiretroviral drugs, including fostemsavir and maraviroc (Muccini et al., 2022; Rose et al., 2022). A recent article described the case of a heavily treatment-experienced patient with a pan-resistant HIV-1 infection (reduced susceptibility to NRTIs, NNRTIs, PIs and INSTIs) who achieved virological control after introducing ibalizumab in association with recycling enfuvirtide and an optimized background regimen (Canetti et al., 2022), confirming that IBA represents an important advance in the management of multidrug-resistant HIV-1 infection.

### Leronlimab (Pro 140)-long-acting CCR5 antagonist

Leronlimab (PRO 140), a humanized IgG4 monoclonal antibody (mAb), acts as an HIV-1 CCR5 antagonist; it binds to hydrophilic extracellular domains on CCR5, inhibiting HIV-1 viral entry in a competitive manner (Thompson, 2018).

The safety and effectiveness of leronlimab is currently, evaluated in phase IIb/III clinical trials. In CD 02 trial (PRO 140_CD 02; NCT02483078), leronlimab was tested in treatment-experienced patients with multidrug resistant HIV. In the first week, participants received 350 mg of PRO 140 subcutaneously or placebo together with their regular medication and 1 week later, they received PRO 140 with an optimized regimen. The primary endpoint of the trial was achieved: patients in the PRO 140 arm showed a statistically significant reduction in HIV RNA viral load of greater than 0.5log from baseline versus patients in the placebo arm.^4^ A recent report showed that all CCR5-tropic strains were fully susceptible to PRO 140 in a group of heavily treatment-experienced HIV-1-positive patients harboring 4-class drug-resistance to NRTIs, NNRTIs, PIs, and InSTIs; current exposure to maraviroc (68% of the participants) was not associated with different PRO 140 activity (Rusconi et al., 2022).

CD03 clinical trial has evaluated weekly subcutaneous PRO 140 as monotherapy maintenance in patients with viral suppression (PRO 140_CD03; NCT02859961). The rate of virological failure was lower in the group of participants receiving 700 mg dose comparing with 525 mg and 350 mg dose groups (13.8, 33 and 65.9%, respectively); no evidence of treatment-emergent resistance to leronlimab was observed. A report published on March 31, 2022, showed that weekly injections of Leronlimab (700 mg dose) maintained viral suppression in the five HIV+ participants for over seven years (ClinicalTrials.gov NCT02175680 and NCT02355184) (Chang et al., 2022).

### UB-421-CD4 attachment inhibitor

UB-421, a humanized IgG1 monoclonal antibody (mAb), acts as a CD4 attachment inhibitor. UB-421 targets domain 1 of CD4 receptor, blocking HIV entry into the cells *via* a competitive binding inhibition (Wang et al., 2019).

Several collaborative studies showed the efficacy of UB-421 against HIV strains resistant to broadly neutralizing HIV antibodies, entry inhibitors, and other ARV drugs [ClinicalTrials.gov NCT03164447]. Currently, UB-421 is in phase 2/phase 3 clinical trials for ART substitution (ClinicalTrials.gov NCT03149211), treatment of patients with multi-drug resistant HIV infection (ClinicalTrials.gov NCT04406727), as well as for HIV functional cure (ClinicalTrials.gov NCT04404049).

### Islatravir-nucleoside reverse transcriptase translocation inhibitor

Islatravir (ISL, MK-8591) is an investigational drug belonging to a new class of antiretrovirals called nucleoside reverse transcriptase translocation inhibitors (NRTTIs) (Markowitz and Sarafianos, 2018). The active form of islatravir (islatravir-triphosphate) inhibits HIV reverse transcriptase (RT) by multiple mechanisms, including RT translocation inhibition and delayed chain termination through viral DNA structural changes (Salie et al., 2016). ISL showed potent activity against HIV-1, HIV-2, and HIV viral strains with multi-drug resistant mutations (Wu et al., 2017). Due to an unexpected decline in total lymphocyte and CD4+ T-cell counts, clinical studies of injectable islatravir for treatment and all formulations of islatravir for pre-exposure prophylaxis (PrEP) are on full or partial clinical holds, still, arms testing lower dosage are ongoing (ClinicalTrials.gov NCT03272347; NCT04564547; NCT05052996).

### Lenacapavir-long-acting capsid inhibitor

Lenacapavir, the first-in-class long-acting HIV capsid inhibitor, was approved in August, 2022 in the European Union and in December, 2022 in US, for the treatment of HIV infection, in combination with other antiretroviral(s), in patients with multi-drug resistant HIV who have very limited treatment choices.^5^ Lenacapavir is the only HIV treatment option that can be administered twice-yearly, in a long acting subcutaneous formulation (Dvory-Sobol et al., 2022). The novel long-acting agent targets multiple stages of the HIV life cycle: it binds directly to the HIV capsid protein and interferes with capsid-mediated nuclear import of HIV DNA, virion production and proper capsid core formation (Figure 1; Bester et al., 2020; Link et al., 2020). The approval of lenacapavir was supported by the results of the Phase 2/3, double-blinded, placebo-controlled global multicenter CAPELLA trial, that involved heavily treatment-experienced patients with multi-drug resistant HIV-1 (Segal-Maurer et al., 2022). Eighty three percent of the participants that received lenacapavir in combination with an optimized background regimen achieved viral suppression at week 52; a mean increase in CD4 count of 83 cells/μL was reported. Lenacapavir was well tolerated, no drug-related serious side effects being identified.

#### Resistance

Lenacapavir has no overlapping resistance with any currently approved antiretroviral therapy; additional studies have not shown any preexisting resistance mutations or naturally occurring polymorphisms that reduce viral susceptibility to lenacapavir (Margot et al., 2022).

Another capsid inhibitor-VH4004280- is currently investigated in phase I clinical trial for safety, tolerability, and *pharmacokinetic* properties in HIV-negative volunteers.

### GSK2838232-maturation inhibitor

Maturation inhibitors-a new class of antiretroviral agents in clinical development-disrupt the proteases cleavage of the HIV-1 gag protein immediate precursor, leading to immature, noninfectious virus particles (Sundquist and Krausslich, 2012; Wang et al., 2015).

Bevirimat and BMS-955176 (GSK3532795), the first 2 maturation inhibitors, demonstrated antiviral activity but no efficacy against some polymorphisms in the HIV gag region (Wainberg and Albert, 2010) and had associated side effects in phase 2b clinical studies.^6^

GSK2838232 is a second-generation HIV-1 maturation inhibitor in phase IIa clinical trial evaluation; it allows for once-daily administration boosted with a pharmacoenhancer (DeJesus et al., 2020). GSK2838232 was well tolerated, and generated a significant viral load decrease in the group of participants receiving the highest dosage. GSK2838232 is active against HIV isolates resistant to bevirimat.

Others long-acting HIV maturation inhibitors (GSK3640254 and GSK3739937) are in earlier stages of development (Joshi et al., 2020; Spinner et al., 2022).

## New potential targets for HIV treatment

**Figure 2:**
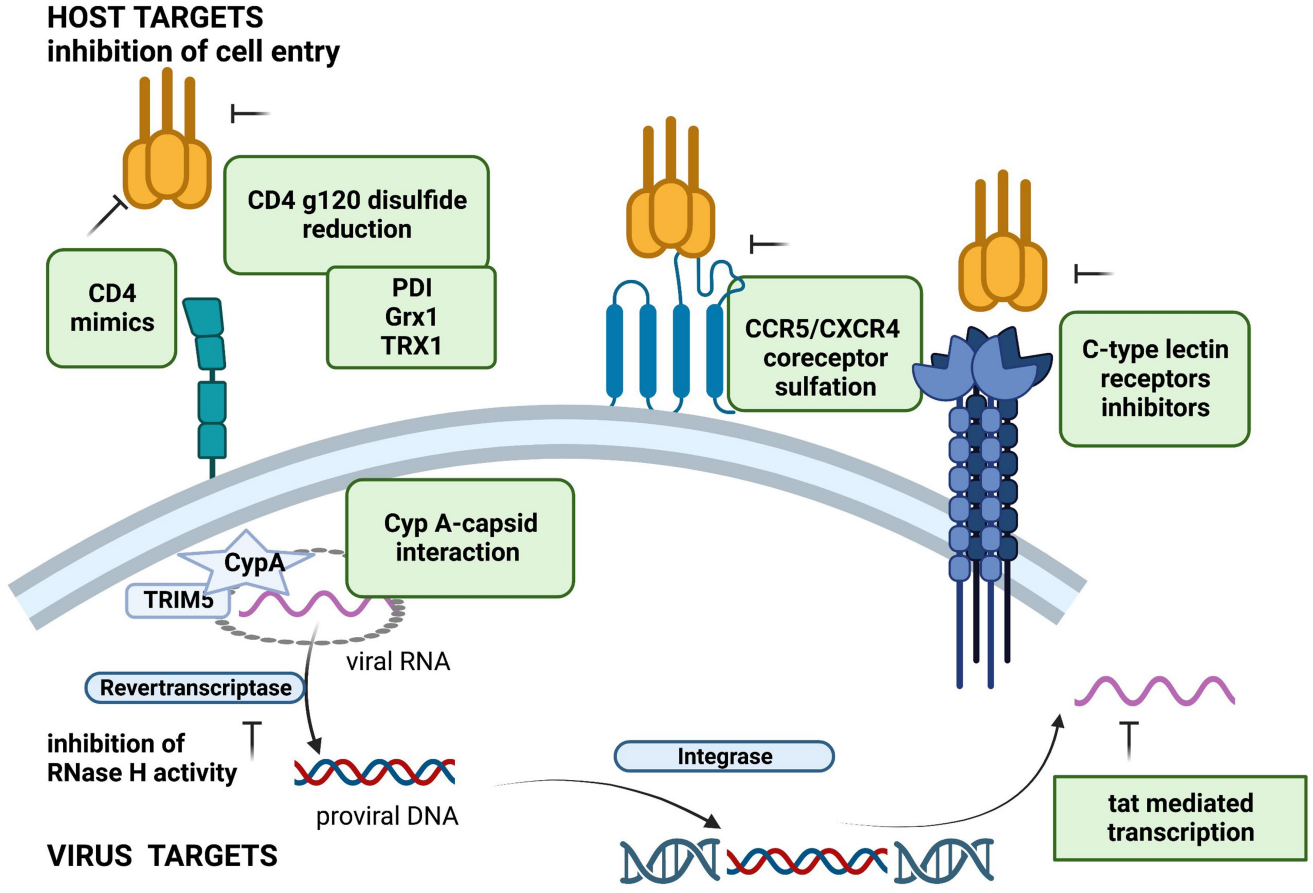
Potential host and viral targets for HIV replication inhibition. Created with BioRender.com, with a license to publish.

To overcome the resistance to HIV-1 antiretroviral therapy, novel drug targets (both virus and host-related) are being investigated (Figure 2).

### Host-related targets

Host factors play an important role in HIV infection (Friedrich et al., 2011). The potential host targets can interfere with HIV entry, replication and spread of HIV infection, and may lead to a higher barrier to antiretroviral drug resistance.

Inhibitors targeting HIV viral entry are among the most attractive targets due to their potential to limit *de novo* infected cells. Several studies showed that HIV-1 entry is dependent on redox control and the compounds that inhibit thiol/disulfide exchange reactions could suppress HIV-1 viral entry (Moolla et al., 2016; Reiser et al., 2016).

#### Protein disulfide isomerase, thioredoxin-1, and galectin-9

Human protein disulfide isomerase (PDI) catalyzes reduction of disulfides in HIV gp120 necessary for virus entry (Markovic et al., 2004). Several PDI inhibitors and anti-PDI antibodies were described, but none has been used therapeutically due to their toxicity (Khan et al., 2011). The interaction of PDI with human protein Galectin-9 regulates the redox environment and enhance T-cell migration and HIV entry (Bi et al., 2011). Moreover, Galectin-9 was reported to be an important mediator of HIV transcription and reactivation of latent HIV in CD4 + T cells and may be explored for novel HIV cure strategies (Abdel-Mohsen et al., 2016; Colomb et al., 2019).

A host protein-oxidoreductase glutaredoxin 1 (Grx1)- was shown to catalyze the reduction of HIV gp120 and CD4 disulfides and its inhition reduced HIV replication (Auwerx et al., 2009). More recent studies reported that the host thioredoxin-1 (Trx1) is also a very efficient reductase of disulfides of gp120 and CD4 *in vitro* and may represent a future host target for HIV-1 treatment (Moolla et al., 2016; Lundberg et al., 2019); inhibition of thioredoxin-1 with anti-Trx1 antibodies, inhibited HIV-1 entry by >80% in cell cultures.

#### CD4 mimics

Several small molecule CD4 mimics have been reported as HIV-1 entry inhibitors, which are blocking the interaction of gp120 with CD4 (Curreli et al., 2016). Recently, novel soluble-type CD4 molecule mimics have been developed with a high anti-HIV activity and synergistic anti-HIV activity with a neutralizing antibody (Kobayakawa et al., 2019; Tsuji et al., 2022).

#### C-type lectin receptors

C-type lectin receptors (CLRs) (galectin-1, DC-SIGN, dendritic cell immunoreceptor-DCIR, etc.), expressed on the surface of dendritic cells (DCs) are binding to HIV gp120 glycoprotein playing an important role in the pathogenesis of HIV infection (Mesman and Geijtenbeek, 2012). Several CLR inhibitors, that modulate protective immune responses and block receptor binding have been described (Varga et al., 2014; Cardinaud et al., 2017; Porkolab et al., 2018). Competitive inhibitions of galectin-1, DC-SIGN and DCIR have shown efficacy in blocking CD4^+^T cells viral infection and HIV propagation, being considered a promising therapeutic target for HIV treatment (Porkolab et al., 2018).

Inhibition of cellular restriction factors is also studied as a potential host target. The human cyclophilin A (CypA) binds HIV-1 viral capsid (CA) and promotes capsid uncoating and viral infectivity (Colgan et al., 1996; Gamble et al., 1996). Disruption of CypA-capsid interactions inhibits HIV-1 replication in cell cultures (Sokolskaja et al., 2004). A recent study showed that Cyclophilin A prevents HIV-1 restriction in lymphocytes by blocking TRIM5α (human tripartite motif-containing protein 5) binding to the viral core (Selyutina et al., 2020). TRIM5α is an antiretroviral restriction factor, that induces premature uncoating, inhibiting reverse transcription and nuclear import (Ganser-Pornillos and Pornillos, 2019). Several compounds were described as HIV-1 assembly and disassembly dual inhibitors, targeting both HIV-1 capsid protein and cyclophilin A (Liu et al., 2016; Obubeid et al., 2022).

### Virus-related targets

Non-conventional viral inhibitors might act on multidrug resistant HIV strains.

#### Inhibitors of RT RNAse H activity

HIV-1 RT possess a ribonuclease activity (RNase H) essential for viral replication (Menéndez-Arias et al., 2017). Two types of RNase H inhibitors have been developed, but none has reached the clinical phase: active site inhibitors, which chelate the two Mg2+ within the active site, and Rnase H allosteric inhibitors (Wang et al., 2018; Xi et al., 2019; Martín-Alonso et al., 2022).

#### Tat inhibitors

HIV-1 transcription factor Tat is required for transcription of the integrated viral genome (Selby et al., 1989). Tat-mediated transcription is conserved and is distinguishable from cellular transcription, being an important therapeutic target against HIV replication and the resistance emergence (Ott et al., 2011; Mousseau and Valente, 2012). The HIV-1 Tat protein regulates the passage from viral latency to active transcription and its inhibition can prevent HIV-1 reactivation and facilitate permanent HIV-1 suppression (Karn, 2011). Several Tat inhibitors have been described, including didehydro-cortistatin A (dCA), that can prevent HIV-1 reactivation from latency (Li et al., 2019; Nchioua et al., 2020) and is studied, together with other latency blocking agents, for the induction of a functional cure. Recently, several 3-oxindole derivatives-with heterocyclic structures have been shown to specifically inhibit the Tat-mediated viral transcription acting on the HIV-1 LTR promoter (Kim et al., 2022). Future strategies focused on agents targeting latent HIV-1 reservoirs are explored to achieve a functional cure.

## Conclusion

Life-long HIV treatment and drug resistance are still a challenge that requires constant efforts to identify new generations of antiretroviral agents. Significant progress has been made for the treatment of HIV drug resistant infection, but new targets are needed for a salvage therapy.

## Author contributions

SR conceived and supervised the review topics. AT wrote the first draft. All authors contributed to the article and approved the submitted version.

## Conflict of interest

The authors declare that the research was conducted in the absence of any commercial or financial relationships that could be construed as a potential conflict of interest.

## Publisher’s note

All claims expressed in this article are solely those of the authors and do not necessarily represent those of their affiliated organizations, or those of the publisher, the editors and the reviewers. Any product that may be evaluated in this article, or claim that may be made by its manufacturer, is not guaranteed or endorsed by the publisher.

## Footnotes

^1^ https://www.unaids.org/sites/default/files/media_asset/JC3032_AIDS_Data_book_2021_En.pdf

^2^ https://www.who.int/publications/i/item/9789240038608

^3^ https://clinicalinfo.hiv.gov/sites/default/files/guidelines/documents/adult-adolescent-arv/guidelines-adult-adolescent-arv.pdf

^4^ https://content.equisolve.net/_0fabb8b784fee97a4a4b390ab5453a6b/cytodyn/db/193/2464/pdf/CytoDyn+-+ASM+2019+Poster+Presentation+re+CD02_Final.pdf

^5^ https://www.ema.europa.eu/en/documents/product-information/sunlenca-epar-product-information_en.pdf

^6^ http://i-base.info/htb/30865
